# Investigations on therapeutic glucocerebrosidases through paired detection with fluorescent activity-based probes

**DOI:** 10.1371/journal.pone.0170268

**Published:** 2017-02-16

**Authors:** Wouter W. Kallemeijn, Saskia Scheij, Sascha Hoogendoorn, Martin D. Witte, Daniela Herrera Moro Chao, Cindy P. A. A. van Roomen, Roelof Ottenhoff, Herman S. Overkleeft, Rolf G. Boot, Johannes M. F. G. Aerts

**Affiliations:** 1 Department of Biochemistry, Leiden Institute of Chemistry, Leiden University, Leiden, The Netherlands; 2 Department of Medical Biochemistry, Academic Medical Center, University of Amsterdam, Amsterdam, The Netherlands; 3 Department of Bio-organic Synthesis, Leiden Institute of Chemistry, Leiden University, Leiden, The Netherlands; Azienda Ospedaliero-Universitaria Santa Maria della Misericordia, ITALY

## Abstract

Deficiency of glucocerebrosidase (GBA) causes Gaucher disease (GD). In the common non-neuronopathic GD type I variant, glucosylceramide accumulates primarily in the lysosomes of visceral macrophages. Supplementing storage cells with lacking enzyme is accomplished *via* chronic intravenous administration of recombinant GBA containing mannose-terminated *N*-linked glycans, mediating the selective uptake by macrophages expressing mannose-binding lectin(s). Two recombinant GBA preparations with distinct *N*-linked glycans are registered in Europe for treatment of type I GD: imiglucerase (Genzyme), contains predominantly Man(3) glycans, and velaglucerase (Shire PLC) Man(9) glycans. Activity-based probes (ABPs) enable fluorescent labeling of recombinant GBA preparations through their covalent attachment to the catalytic nucleophile E340 of GBA. We comparatively studied binding and uptake of ABP-labeled imiglucerase and velaglucerase in isolated dendritic cells, cultured human macrophages and living mice, through simultaneous detection of different GBAs by paired measurements. Uptake of ABP-labeled rGBAs by dendritic cells was comparable, as well as the bio-distribution following equimolar intravenous administration to mice. ABP-labeled rGBAs were recovered largely in liver, white-blood cells, bone marrow and spleen. Lungs, brain and skin, affected tissues in severe GD types II and III, were only poorly supplemented. Small, but significant differences were noted in binding and uptake of rGBAs in cultured human macrophages, in the absence and presence of mannan. Mannan-competed binding and uptake were largest for velaglucerase, when determined with single enzymes or as equimolar mixtures of both enzymes. *Vice versa*, imiglucerase showed more prominent binding and uptake not competed by mannan. Uptake of recombinant GBAs by cultured macrophages seems to involve multiple receptors, including several mannose-binding lectins. Differences among cells from different donors (*n* = 12) were noted, but the same trends were always observed. Our study suggests that further insight in targeting and efficacy of enzyme therapy of individual Gaucher patients could be obtained by the use of recombinant GBA, trace-labeled with an ABP, preferably equipped with an infrared fluorophore or other reporter tag suitable for *in vivo* imaging.

## Introduction

Glucocerebrosidase (GBA) is a retaining β-glucosidase that hydrolyzes the glyco-sphingolipid glucosylceramide in the lysosome [1–3]. Deficiency of GBA activity causes Gaucher disease (GD), the most common inherited lysosomal storage disorder [1–3]. Symptoms include hepatosplenomegaly, cytopenia and osteopenia [4–6], with highly variable manifestations, ranging from the (common) non-neuronopathic GD type I variant to more severe manifestations with lethal neurological complications (GD type II and III) and extreme cases with abnormalities in skin permeability (collodion babies) [4–6].

In type I Gaucher disease, lysosomal accumulation of glucosylceramide is mostly restricted to visceral macrophages, named Gaucher cells [4–10]. Macrophages are primarily affected by the deficiency of GBA, due to high rates of lysosomal breakdown of complex glycosphingolipids following phagocytosis of senescent erythrocytes and apoptotic cells, as well as uptake of lipoproteins [11–15]. Gaucher cells are believed to cause many of the visceral symptoms [4–6] and the prevention and/or removal of these lipid-laden macrophages has been formulated as a rational target for treatment of type I GD [16, 17]. Brady and colleagues developed an elegant approach to specifically supplement visceral macrophages with exogenous GBA [18, 19], exploiting the presence of mannose-binding lectin(s) in this cell-type [20–25]. Isolated placental GBA, containing four *N-*linked glycans, largely of the complex type, was enzymatically modified to expose terminal mannose-moieties (alglucerase, Ceredase^TM^, Genzyme) [26, 27]. Later on, human GBA was produced in CHO cells [28, 29], and comparably modified [28]. The recombinant enzyme, named imiglucerase (Cerezyme^TM^, Genzyme), contains largely Man(3) *N*-linked glycans [30–32]. Enzyme therapy with imiglucerase results in impressive clinical responses in type I GD patients [33, 34] and is a worldwide registered therapy for this disorder.

More recently, an alternative recombinant GBA has been produced by Shire PLC [35], using the method of gene-activation, resulting in an enzyme (velaglucerase, VPRIV^TM^) with largely Man(9) *N*-linked glycans [30, 32]. The latter enzyme recently received orphan drug status in Europe and the USA, based on the successful outcome of clinical trials with type I GD patients [35]. Recently, another recombinant GBA, produced in plant cells (taliglucerase, Elelyso^TM^, Protalix/Pfizer) [30, 32, 36, 37], was registered in the USA and Europe, but this enzyme received no orphan drug status in Europe and consequently its use on this continent is restricted [38].

The availability in Europe of two comparably costly, recombinant GBA preparations, differing in *N*-linked glycan composition essential for the targeting to macrophages, has raised considerable interest into their therapeutic efficacy. To this date, there are no clinical trials yet described which directly compare the various effects and outcomes of imiglucerase and velaglucerase therapy, at equal dose and with matched patients. Switch-over of imiglucerase to velaglucerase therapy has taken place as a consequence of the global shortage in imiglucerase in 2009, stemming from problems with production [39]. Type I GD patients switching from imiglucerase to velaglucerase showed no associated changes in clinical responses and parameters as well as biomarkers [40, 41]. There are a few literature reports describing comparative studies on imiglucerase and velaglucerase by means of crystallography, analytical chemistry and enzymology [30, 32, 42]. Experiments were also designed to examine binding and uptake of both recombinant GBA preparations using cultured cell models [42]. Finally, bio-distribution of intravenously injected rGBAs was studied in mice [43–46] but no comparative data for both enzymes have been reported.

**Fig 1:**
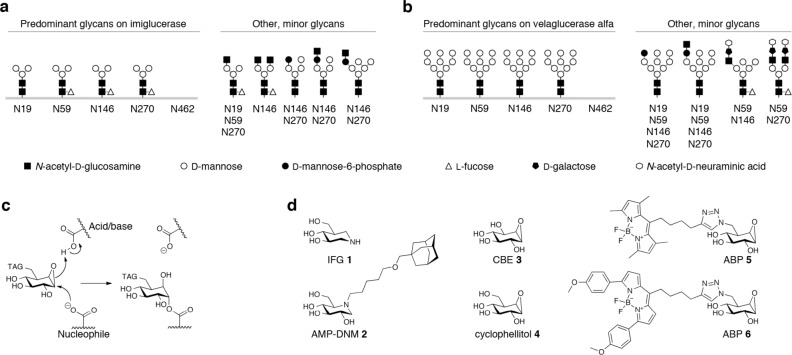
Background. *N*-linked glycan composition of recombinant GBAs imiglucerase (**a**) and velaglucerase (**b**) according to refs[30, 32]. (**c**) Mechanism of irreversible inhibition by β-epoxide ring-opening. (**d**) Structures of isofagomine (IFG **1**), AMP-DNM **2**, conduritol β-epoxide (CBE **3**), cyclophellitol **4**, and green− and red-fluorescent β-epoxide ABPs **5** and **6** (MDW933, MDW941)[48].

The documented differences in structure of *N*-linked glycans of imiglucerase and velaglucerase (*predominantly Man(3) versus Man(9) structures*, *respectively*, *see* Fig 1A) indicate that significant differences might exist between the two rGBAs, regarding their targeting to macrophages and consequently their bio-distribution and efficacy. There has only been a single study attempting to visualize the fate of intravenously administered therapeutic GBA in a Gaucher patient and healthy subject [47]. In this seminal investigation, placental, mannose-terminated GBA (alglucerase) was radio-labeled with ^123^I, which randomly modifies tyrosine residues. Gamma scintigraphy was used to visualize the bodily distribution of the therapeutic enzyme. This approach, despite its intrinsic low resolution, showed convincing accumulation of GBA in spleen, liver and bone marrow [47]. At present, comparable information on the widely applied recombinant enzymes imiglucerase and velaglucerase is lacking. More insight in bio-distribution and targeting to visceral macrophages of these rGBAs is urgently needed given the high costs of enzyme therapy for Gaucher disease.

We recently developed a new technology to visualize GBA molecules employing activity-based probes (ABPs) [48]. We demonstrated that fluorescent BODIPY-containing cyclophellitol β-epoxide, hijacking the catalytic double-displacement mechanism of GBA, forms an irreversible inhibitor-nucleophile E340 adduct (Fig 1B). Various ABPs have meanwhile been designed including probes with a green− and red fluorophore, respectively green β-epoxide ABP **5** and red β-epoxide ABP **6** (Fig 1C) [48]. The covalent labeling of GBA *via* E340 is highly specific and detection of fluorescently labeled enzyme is ultra-sensitive (*detection limit ~20 attomol*, *or 10*^*−18*^ *mol*). The availability of these ABPs allows a defined and subtle labeling of GBA molecules [48] and analysis of their fate following administration to cells or after intravenous infusion in mice.

Here we report on a comparative investigation of imiglucerase and velaglucerase labeled with ABPs that fluoresce at different wavelengths. We demonstrate that both enzymes are very similar in catalytic features while differing in *N*-linked glycan composition. Binding and uptake of ABP-labeled imiglucerase and velaglucerase in isolated dendritic cells, cultured human macrophages and living mice was investigated and the outcome of these investigations is presented. ABP-labeled imiglucerase and velaglucerase, administered as single enzymes or as equimolar mixture, were similarly taken up by murine dendritic cells and showed a comparable bodily distribution following intravenous administration to living mice. No striking differences were noted between the two rGBAs in the targeting of key organs in the pathology of type I GD. Enzymes were recovered largely in liver, white-blood cells, bone marrow and spleen. More detailed investigations on binding and uptake by macrophages were performed using cultured macrophages, derived from human peripheral blood monocytes. Subtle differences were noted between the two recombinant enzymes. Our study firstly demonstrates the value of ABPs to comparatively investigate two rGBAs with respect to their uptake by macrophages and bodily distribution following intravenous infusion of mice.

## Materials and methods

### General methods

ABPs were synthesized as described earlier [48]. Chemicals were obtained from Sigma-Aldrich if not otherwise indicated. Recombinant GBA was purchased from Genzyme (imiglucerase; imi) and Shire PLC (velaglucerase; vela). Monoclonal anti-human GBA antibody 8E4 was produced from hybridoma cells as described earlier [49]. Immature dendritic cells were a kind gift from the Biopharmaceutics (LACDR) Department of Leiden University. Cell lines were cultured in HAMF12-DMEM medium (Invitrogen) supplied with 10% FBS. Buffy-coats were purchased from Sanquin Bloodbank (Amsterdam).

### Ethics

The appropriate ethics committees for animal experiments approved this study and all experimental procedures including those involved with the isolation of bone marrow derived dendritic cells (Leiden University, Leiden, The Netherlands) and the procedures involved with *in vivo* biodistribution studies (Academic Medical Center, University of Amsterdam, The Netherlands).

### Enzyme activity assays

The activity of GBA was assayed at 37°C by incubating with 3.75 mM 4-methylumbelliferyl-β-d-glucopyranoside (4MU-β-d-Glc) as substrate in 150 mM McIlvaine buffer, pH 5.2, supplemented with 0.1% (w/v) BSA, 0.2% (w/v) sodium taurocholate, and 0.1% (v/v) Triton X-100. Determination of reaction constants was performed by incubating GBA with 0−4 mM 4MU-β-d-Glc and fitting results to a Michaelis-Menten function. Effect of pH on enzymatic β-glucosidase activity was analyzed by pre-incubating GBA in pH 2−9 for 30 min at 37°C, whereafter GBA activity as assayed with 3.75 mM 4MU-β-d-Glc at the same pH. Time-dependent decay of GBA β-glucosidase activity towards 4MU-β-d-Glc was measured by firstly incubating the enzyme at pH 5.2 or 7.4, whereafter GBA activity was assayed with 3.75 mM 4MU-β-d-Glc at pH 5.2. Inhibitory potency of IFG **1**, AMP-DNM **2**, conduritol β-epoxide (CBE **3**), cyclophellitol **4**, and β-epoxide type ABP **5** (MDW933) and ABP **6** (MDW941) was determined by pre-incubating GBA with a range of inhibitor concentrations for 30 min at pH 3–9, where after the residual GBA β-glucosidase activity was measured by addition of 3.75 mM 4MU-β-d-Glc at pH 5.2. After stopping each substrate reaction with excess NaOH-glycine (pH 10.3), fluorescence was measured with a fluorimeter LS55 (Perkin Elmer) using λ_EX_ 366 nm and λ_EM_ 445 nm.

### ABP-labeled rGBA preparations

Concentrations of imiglucerase (imi) and velaglucerase (vela) preparations were determined with standard 4MU-β-d-Glc assay conditions at pH 5.2 and subsequently labeled for 50% or 90% with either green fluorescent β-epoxide ABP **5** or red fluorescent β-epoxide ABP **6** in 150 mM McIlvaine buffer, pH 5.2 (supplemented with 0.2% (w/v) sodium taurocholate, 0.1% (v/v) Triton X-100) for 1 h at 37°C. Unbound β-epoxide ABPs **5** and **6** were removed by washing thrice with the aforementioned McIlvaine buffer over 30 kDa cut-off filters. Equimolar mixes were prepared of apo: green ABP **5** or red **6**-labeled imiglucerase, apo: red ABP **6**-labeled velaglucerase and green ABP **5**-labeled imiglucerase: red ABP **6**-labeled velaglucerase. Total inactivation of each enzyme preparation was quantified by 4MU-β-d-Glc assays, with and without pre-incubations with spikes of 12.5 μL 10 nM imiglucerase. In parallel, samples were denatured with 5× Laemmli buffer (50% (v/v) 1M Tris-HCl, pH 6.8, 50% (v/v) 100% glycerol, 10% (w/v) DTT, 10% (w/v) SDS, 0.01% (w/v) bromophenol blue), boiled for 4 min at 100°C, and separated by electrophoresis on 7.5% (w/v) SDS-PAGE gel running continuously at 90 V [48, 50]. Wet slab gels were scanned on fluorescence using the Typhoon Variable Mode Imager (Amersham Biosciences) using λ_EX_ 488 nm and λ_EM_ 520 nm (band pass filter 40 nm) for green fluorescent β-epoxide ABP **5** and λ_EX_ 532 nm and λ_EM_ 610 nM (band pass filter 30 nm) for red fluorescent β-epoxide ABP **6**. ABP-emitted fluorescence was quantified using ImageJ software (NIH, Bethesda, MD, USA), and verified in-gel by presence of 50 fmol equimolar green β-epoxide ABP **5**– and red **6**-labeled imiglucerase.

### Immunoprecipitation

Mouse α-human GBA 8E4 [49] was immobilized on Sepharose-ProtA beads in PBS for 1 h at room temperature (RT) while rotating at 30 rpm, thereafter washed thrice by centrifugation for 3 min at 500 rpm and rinsed with PBS for 10 min. Samples of 10 μL were immunoprecipitated using 50 μL 8E4-Sepharose-ProtA beads for 1 h at RT, thereafter washed thrice by centrifugation for 3 min at 500 rpm and rinsed with PBS for 10 min. Immunoprecipitant and first supernatant were separated by SDS-PAGE and ABP-labeled proteins were visualized by fluorescence scanning.

### Deglycosylation

ABP-labeled proteins were digested with PNGase-F or endo-H, following manufacturers’ instructions (New England Biolabs). After deglycosylation, samples were denatured, separated by SDS-PAGE and ABP-labeled proteins were visualized by fluorescence scanning.

### One/two dimensional SDS-PAGE, gel staining and densitometry

ABP-labeled GBA was precipitated by TCA, re-hydrated (30 mM Tris, 7.7 M urea, 2.2 M thiourea, pH 8.9, 4% (w/v) CHAPS, 0.5% (v/v) Destreak agent and 2% (v/v) IPG buffer), soaked overnight into pH 3−10 nonlinear strips (BioRad). Iso-electric focusing with standard program (0.1 min at 50 V; 30 min to 200 V; 30 min at 200 V; 30 min to 400 V; 30 min at 400 V; 30 min to 600 V; 30 min at 600 V; 60 min to 3,500 V; 240 min at 3,500 V; 10 min to 200 V; hold at 200 V, on Protean IEF (BioRad)) and proteins subsequently separated by SDS-PAGE. One-dimensional SDS-PAGE followed by fluorescent scanning and Coomassie Brilliant Blue (CBB) staining was exactly performed as described earlier [50]. Fluorescence controls were either based on excess imiglucerase labeled with 50 fmol green β-epoxide ABP **5** and red **6**, denatured, mixed and loaded on gel as control standard, or based on excess imiglucerase labeled with 100 fmol green β-epoxide ABP **5** and an equimolar amount of velaglucerase labeled with 100 fmol red **6**, denatured, mixed and loaded on gel as control standard.

### Bio-distribution study of ABPs in living mice

C57Bl/6J mice were obtained from Charles River (Wilmington, MA, USA) and fed a commercially available lab diet (CRM(E), Special Diet Services, UK). Twelve male C57Bl/6J mice were intravenously injected by tail-vein injection with PBS vehicle or 1 nmol green ABP **5**-labeled imiglucerase and 1 nmol red ABP **6**-labeled velaglucerase. After 0–4 days the mice were anesthetized with FFM mix (25/25/50 fentanyl-citrate/midazalam/sterile H_2_O) and EDTA-blood was collected from the tail-vein. Mice were then perfused *via* the heart into the aortic root with PBS, flowing at 3.0 mL min^−1^ for 3 min by using a syringe pump (Harvard apparatus, Holliston, MA, USA), whereafter organs were collected, directly snap-frozen in liquid nitrogen. Samples were stored at –80°C until homogenization in 1:5 ratio of tissue-weight to volume of 25 mM potassium phosphate buffer (pH 6.5, 0.1% (v/v) Triton X-100 and protease inhibitor cocktail (Roche)). Standard tissue homogenization carried out with the organs: brain, liver, spleen, lungs, thymus, kidney, testis, epididymis, stomach, pancreas, duodenum, brown− and epididymal fat. Homogenization occurred with 250 μL RNAse-free glass beads in 2 mL screw-cap Eppendorf tubes, samples were crushed with a TissueLyzer set at 6 m s^−1^ for 20 s, thrice, while samples were chilled on ice-slush between runs for 2 min. Lysates were isolated from glass beads by pipetting into sterile Safe-Lock Eppendorf tubes. After homogenization of adipose tissues, samples centrifuged for 10 min at 10,000 rpm at 4°C and bottom layer of lysate was pipetted into sterile Safe-Lock Eppendorf tubes. The top, lipid-rich layer was isolated and stored separately. Other organs (eye, skin, heart, calf muscle) were first manually cut with a sterile scalpel and subsequently homogenized by sonication at 50% power, 50% amplitude for 5 s, thrice while samples were identically chilled as decribed, *vide supra*. Small isolates, *i*.*e*. white blood cells and bone marrow were directly sonicated as described, *vide supra*. All homogenates were then aliquoted and directly snap-frozen in liquid nitrogen, or immediately analyzed for protein concentration (BCA kit, Pierce), *in vitro* ABP labeling and fluorescence scanning of SDS-PAGE slab-gels. ABP-emitted fluorescence was quantified using ImageJ software (NIH, Bethesda, MD, USA), and verified in-gel by presence of 50 fmol equimolar green β-epoxide ABP **5**– and red **6**-labeled imiglucerase, and calculated values were corrected for CBB staining intensity.

### Culturing of primary cells

C57Bl/6 mice were deeply anesthetized (see above), tibiae and femurs were collected and bone marrow of tibiae was flushed out with PBS. Then, immature dendritic cells were grown in dendritic cell selection medium (2:1 (v/v) IMDM with granulocyte-macrophage colony stimulating factor (GM-CSF), containing 8% (v/v) FCS, penicillin/streptomycin (100 U mL^–1^), glutamax (2 mM) and β-mercaptoethanol (20 μM) with 5% (v/v) CO_2_ at 37°C, and sub-cultured every 2–3 d.

### Confocal fluorescence microscopy

Immature bone marrow derived dendritic cells cultured 10–12 d *ex vivo* were seeded at 30–75 × 10^4^ cells/well into sterile LabTek II 4– or 8-chamber borosilicate cover-glass systems (Fisher Emergo). After 2 h, cells were attached and pre-incubated with 300 μM CBE **3** in absence or presence of 3 μg μL^−1^ mannan for 1 h. Hereafter, cells were incubated with 50 nM green ABP **5**– or red **6**-labeled imiglucerase, velaglucerase, or both labeled with opposite ABPs (equimolar, 1:1) for 2 h. The ABP-labeling of aforementioned rGBA stocks was 50% (50% remained *apo*). Cells were washed twice with PBS and imaged (*live cells*), before being fixed with 4% (w/v) *p*-formaldehyde in PBS, then washed thrice with PBS and finally nuclei were stained with Draq5 (Fisher Scientific) (*fixed cells*). Imaging was performed with a Leica TCS SPE confocal microscope, using GFP, dsRed and Cy5 filter settings (λ_EX_ 488, 532, 635 nm, respectively) with optimized detection range to exclude bleed-through of dye signal in different channels. Micrographs were taken sequentially and laser settings, pinhole and PMT were kept constant in each experiment.

### Isolation and maturation of macrophages

Buffy-coats were diluted into PBS supplemented with 0.1% (w/v) BSA and heparin, subsequently layered on top of Lymphoprep gradient (Stemcell Technologies) and centrifuged at 1,000×g for 15 min at RT. After washing the PBMC pellets with PBS supplemented with 0.1% (w/v) BSA, cells were centrifuged at 750×g for 10 min at RT, rinsed and repeated at 500×g for 5 min. Hereafter, pellet is washed with aforementioned PBS, and centrifuged at 250×g for 10 min at RT. Then, monocytes are separated on a Percoll gradient. Pellet is resuspended in 2.5 mL 60% (w/v) SIP and then layered with 5 mL 45% (w/v) SIP and 2.0 mL 34% (w/v) SIP), and centrifuged at 1750×g for 45 min at RT. Upper interface containing monocytes is washed thrice with aforementioned PBS, centrifuged at 500×g for 10 min and then twice at 500×g for 5 minutes. Cell fraction was then resuspended in RPMI with 1% (w/v) human serum, monocytes counted with tryphan blue solution and 10^6^ monocytes were seeded per well. After 1 h at 37°C and 5% (v/v) CO_2_, non-adhering non-monocyte cells were washed away with aforementioned PBS and adhered monocytes then were cultured in RPMI with 10% (v/v) human serum for 7 days prior to experiment initiation.

### Initial binding and uptake assays with cultured cells

Monocyte-derived macrophages were incubated with 100 nM green β-epoxide ABP **5**– or red **6**-labeled imiglucerase for 0–180 min whilst at 18°C, to block endocytosis, or at 37°C, to allow endocytosis. At different time-points, the medium was removed; cells were washed thrice with ice-cold PBS and subsequently lysed by scraping in 25 mM potassium phosphate buffer (pH 6.5, 0.1% (v/v) Triton X-100 and protease inhibitor cocktail (Roche)). After determination of the protein concentration, samples were denatured, separated by SDS-PAGE and ABP-labeled proteins were visualized by fluorescence scanning. ABP-emitted fluorescence was quantified using ImageJ software (NIH, Bethesda, MD, USA), and verified in-gel by presence of 50 fmol equimolar green β-epoxide ABP **5**– and red **6**-labeled imiglucerase, and calculated values were corrected for CBB staining intensity.

### Cell binding assays

First, monocyte-derived macrophages were incubated with 300 μM CBE **3** for 2 h in medium and washed with PBS. Hereafter, the cells were pre-incubated for 15 min at 18°C in the presence or absence of 10 μg μL^−1^ mannan and subsequently received 0–2,500 nM *apo* or ABP-labeled GBA enzyme preparation for 30 minutes at 18°C. Hereafter medium was removed, cells were washed thrice with ice-cold PBS and subsequently lysed by scraping in 25 mM potassium phosphate buffer (pH 6.5, 0.1% (v/v) Triton X-100 and protease inhibitor cocktail (Roche)). ABP-emitted fluorescence was quantified using ImageJ software (NIH, Bethesda, MD, USA), and verified in-gel by presence of 50 fmol equimolar green β-epoxide ABP **5**– and red **6**-labeled imiglucerase, and calculated values were corrected for CBB staining intensity. For each separate donor, the apparent *K*_*D*_ and *B*_*MAX*_ involved in surface binding of each rGBA was calculated by fitting the equation *rGBA*_*surface-bound*_ *= B*_*MAX*_*[rGBA*_*input*_*] / K*_*D*_*+[rGBA*_*input*_*]* to the employed input and recovered quantity of rGBA, in the presence or absence of mannan. Mannan-competed binding was calculated by subtracting the fitted curve of when mannan was present from the fitted curve of when mannan was absent. Ratios were calculated between *K*_*D*_ and *B*_*MAX*_ for imiglucerase and velaglucerase, per individual donor.

### Cellular uptake assays

Uptake experiments were performed identically to binding assays, except incubations were performed at 37°C. For each donor, apparent *K*_*D*_ and *B*_*MAX*_ involved in cellular uptake and surface binding of each rGBA was calculated by fitting the equation *rGBA*_*surface-bound*_ *= B*_*MAX*_*[rGBA*_*input*_*] / K*_*D*_*+[rGBA*_*input*_*]* to the employed input and recovered quantity of rGBA, in the presence or absence of mannan. Cellular uptake of each rGBA was calculated by subtracting the curves fitted for the binding only, with mannan-competed binding determined by subtracting the fitted curve of when mannan was present from the fitted curve of when mannan was absent. Ratios were calculated between *K*_*D*_ and *B*_*MAX*_ for imiglucerase and velaglucerase, per individual donor.

## Results

### ABP-labeling and characterization of imiglucerase and velaglucerase *in vitro*

**Fig 2:**
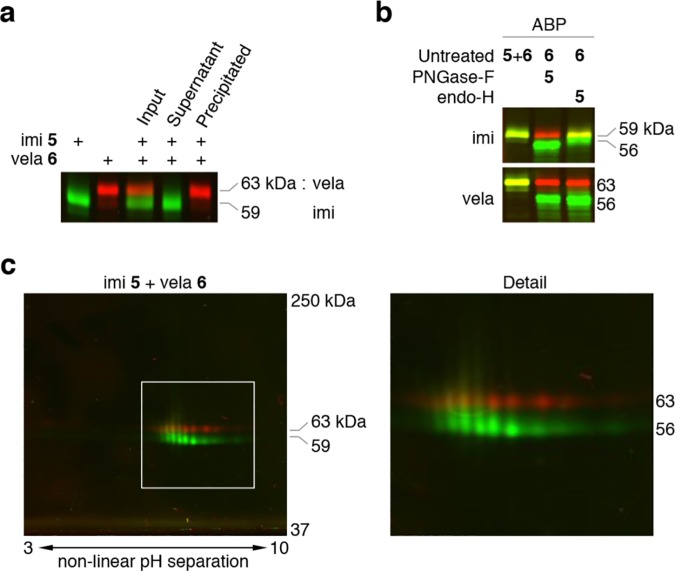
Labeling of imiglucerase and velaglucerase with ABPs. (**a**) From *left* to *right*: visualized after SDS-PAGE, imiglucerase labeled with green ABP **5**; velaglucerase with red ABP **6**; a mixture of both pre-labeled rGBAs; supernatant of mixture immunoprecipitated with monoclonal 8E4; immunoprecipitate. (**b**) Enzymatic deglycosylation of ABP **5**-labeled rGBA by PNGase-F and endo-H. *Top panel* shows imiglucerase, *bottom panel* velaglucerase. Every lane contained red ABP **6**-labeled rGBA. From *left* to *right*: untreated rGBA labeled with green ABP **5** (*yellow color due to overlay with red ABP* ***6****-labeled corresponding enzyme*); PNGase-F treated rGBA labeled with green ABP **5** and endo-H treated rGBA labeled with green ABP **5**. (**c**) Two-dimensional gel electrophoresis of equimolar mixture of green ABP **5**-labeled imiglucerase and red ABP **6**-labeled velaglucerase (Detail; *right*).

Consistent with earlier literature reports [30, 32], imiglucerase and velaglucerase preparations were found to be quite similar in enzymatic features. Using 4-methylumbelliferyl β-d-glucopyranoside as artificial substrate, imiglucerase and velaglucerase showed a comparable V_max_ (50.68 and 50.22 nmol min^−1^ μg^−1^, respectively), K_m_ (1.828 and 1.837 mM, respectively) and pH-dependence with an optimal activity at 5.2 (*see* S1 Fig). The two enzymes were also comparably inactivated by exposure to pH 7.4, *i*.*e*. the pH of whole blood. Both preparations prove equally sensitive to inhibition by the reversible inhibitors isofagomine (IFG **1**) and AMP-DNM **2**, and similarly inactivated by the irreversible inhibitors conduritol β-epoxide (CBE **3**) and cyclophellitol **4**, as assessed at different pH values (*see* S1 Fig). Importantly, the irreversible inactivation of imiglucerase and velaglucerase by cyclophellitol β-epoxide type ABP **5** was identical. Likewise, β-epoxide ABP **6** comparably inactivated imiglucerase and velaglucerase (S1 Fig). No differences were noted in reactivity of the two rGBAs with the two ABPs. The inactivation with both ABPs was complete for both enzymes. Since inactivation of GBA by ABPs implies covalent labeling of the nucleophile E340, we examined the formation of ABP-labeled enzyme by subjecting rGBAs that had been incubated with β-epoxide ABPs to SDS-PAGE. Fluorescence scanning of the slab gel showed the presence of ABP-labeled imiglucerase and velaglucerase (Fig 2A, *first two lanes*).

Imiglucerase and velaglucerase differ in apparent molecular weight with SDS-PAGE due their difference in *N*-linked glycans, *i*.*e*. predominantly Man(3) and Man(9), respectively. Even when mixed in a single sample, imiglucerase and velaglucerase are clearly separated by SDS-PAGE in 7.5% polyacrylamide gels (Fig 2A, *e*.*g*. *middle lane*).

Imiglucerase contains an arginine at position 496 instead of a histidine residue as in velaglucerase and wild-type GBA [28–32]. Consequently, imiglucerase does not cross react with the monoclonal antibody 8E4 [48, 51]. When equal amounts (500 fmol) of imiglucerase and velaglucerase were labeled with excess green fluorescent β-epoxide ABP **5** or red fluorescent β-epoxide ABP **6**, immunoprecipitation with 8E4 antibody led to selective removal of velaglucerase (Fig 2A, *last two lanes*).

Enzymatic removal of *N*-linked glycans from imiglucerase and velaglucerase with PNGase-F, results in reduction of molecular weight to ~56 kDa, both for imiglucerase and velaglucerase (Fig 2B, *last lane*). Removal of the high mannose-core by endo-H changed the molecular weight of imiglucerase to approximately 58−59 kDa, in contrast to that of 56 kDa shown by endo-H digested velaglucerase (Fig 2B, *middle lane*). Green ABP **5**-labeled imiglucerase and red ABP **6**-labeled velaglucerase were mixed equally (1 picomol) and subjected to 2D gel electrophoresis (Fig 2C). Subtle differences in isoforms of imiglucerase and velaglucerase become apparent with this analysis. Most likely the distinct isoform profiles of both enzymes reflect differences in glycan composition, which might influence uptake and/or stability. Swapping β-epoxide ABP **5** for **6** (and *vice versa*) for both enzymes did not alter the isoforms or 2D gel electrophoresis patterns (*data not shown*).

### Uptake of ABP-labeled imiglucerase and velaglucerase by murine dendritic cells

**Fig 3:**
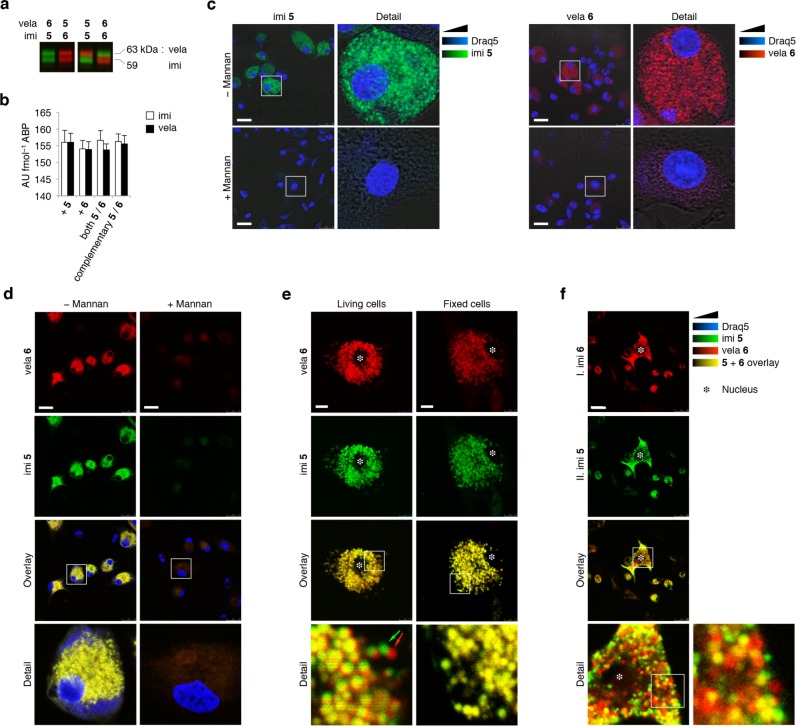
Uptake of ABP-labeled rGBAs by dendritic cells. (**a**) ABP **5**− and **6**-labeled imiglucerase and velaglucerase. (**b**) Arbitrary units of fluorescence (AU) quantified per fmol ABP-labeling of imiglucerase (*open columns*) and velaglucerase (*closed columns*). (**c**) CSLM micrographs of fixed murine bone marrow-derived immature dendritic cells after 30 min uptake of green ABP **5**-labeled imi and red ABP **6**-labeled vela in absence (*top row*) and presence of 1 μg/μL mannan (*bottom row*). Nuclei in blue (Draq5 staining). Micrographs were merged with bright-field microscopic image. Scale-bar represents 25 μm. (**d**) Uptake of equimolar mixture of green ABP **5**-labeled imi and red ABP **6**-labeled vela without and with 1 μg/μL mannan (*left* and *right column*, respectively), analyzed when fixed. Equal green and red fluorescence yields yellow overlay. Scale-bar represents 25 μm. (**e**) Dynamic endocytosis of equimolar mixture of ABP **5**-labeled imi and ABP **6**-labeled vela as registered in living cells (*left column*). Arrows show shift in red ABP **6** and green ABP **5**-labeled rGBA visualized at different time-point. *Right column*: identical experiment but with fixed cells prior to fluorescence acquisition. Asterisk depicts nucleus, scale-bar represents 7.5 μm. (**f**) Dynamic uptake of ABP-labeled rGBA, visualized by primary 75-minute pulse of red ABP **6**-labeled imi followed by washing and secondary 75-minute pulse of green ABP **5**-labeled imi. Asterisk depicts nucleus, scale-bar represents 25 μm.

Batches of equal amounts of imiglucerase and velaglucerase were covalently labeled with green β-epoxide ABP **5** and red ABP **6** while avoiding the presence of excess free β-epoxide ABP (Fig 3A). Quantification of ABP-emitted fluorescence resulted in 155.8 ± 0.6 arbitrary fluorescence units (AU) per fmol β-epoxide ABP **5** or **6**, for both imiglucerase or velaglucerase (Fig 3B).

Next, murine bone marrow-derived dendritic cells, known to contain mannose-binding lectin(s), were treated with green β-epoxide ABP **5**-labeled imiglucerase or red ABP **6**-labeled velaglucerase, in the absence and presence of mannan, which is a competitor for mannose-binding lectin-dependent uptake (Fig 3C). After thorough washing and subsequent fixation, the subcellular distribution of fluorescent enzyme was determined. Both enzymes were avidly taken up in the dendritic cells in the absence of mannan (Fig 3C, *top row*) with no apparent differences in subcellular localization. The presence of mannan completely inhibited uptake of either ABP-labeled rGBA (Fig 3C, *bottom row*). The same findings were made for red ABP **6**-labeled imiglucerase and green β-epoxide ABP **5**− and red ABP **6-**labeled velaglucerase (*data not shown*).

In a similar experiment, dendritic cells were incubated with an equimolar mixture of green β-epoxide ABP **5**-labeled imiglucerase and red ABP **6**-labeled velaglucerase, with a comparable outcome (Fig 3D, *left column*). Both green and red rGBAs were taken up with approximately similar efficiency, resulting in a yellow overlay. Uptake of the rGBAs into the dendritic cells was completely competed by mannan (Fig 3D, *right column*).

Live-cell imaging, thus without fixation, suggested uptake of ABP-labeled imiglucerase and velaglucerase occurs in the same vesicle (Fig 3E, *left column*). While acquiring the micrographs, firstly red ABP **6**-emitted fluorescence was detected; the apparent shifts of red and green labeled structures in the two stills suggest movement of vesicles in the period of time between the red and green fluorescence data acquisition (Fig 3E, *detail*, *see bottom left and arrows*). When fixing the cells with 4% (w/v) *p*-formaldehyde, the earlier observed shifts between red and green fluorescence were absent: labeled imiglucerase and velaglucerase co-localized completely (Fig 3E, *right column*). The dynamic process of rGBA internalization by endocytosis was visualized by a pulse-pulse experiment, consisting of firstly exposing cells to ABP **6**-labeled imiglucerase for 75 min, followed by washing and exposure to fresh medium containing ABP **5**-labeled imiglucerase for 75 min. As can be seen in Fig 3F, vesicles can be observed containing predominantly ABP **5** or **6**-labeled imiglucerase, or both. After 150 min, the vesicles positive in ABP **6**-labeled imiglucerase tend to be in closer proximity to the nucleus compared to those containing predominantly ABP **5**-labeled imiglucerase, (Fig 3F, *detail*).

### Binding of ABP-labeled rGBAs by human monocyte-derived macrophages

To characterize the targeting of rGBAs to macrophages and their subsequent uptake of rGBAs in more detail, we performed a series of experiments with macrophages derived from human peripheral blood monocytes (detailed data for cells of individual donors in such experiments are provided in S3–S6 Figs). For these experiments we used rGBA concentrations in the same range as expected to occur in type I GD patients during infusion. Patients usually receive doses of 30–60 IU kg^−1^ bodyweight during an infusion of one hour. If all of the administered enzyme would accumulate in plasma, this would lead to concentrations of 40–80 nM. In reality, the steady-state concentration during the infusion is usually much lower, in the range of 5–20 nM, as the result of ongoing uptake into cells and slow infusion (Aerts *et al*., *unpublished observations*).

**Fig 4:**
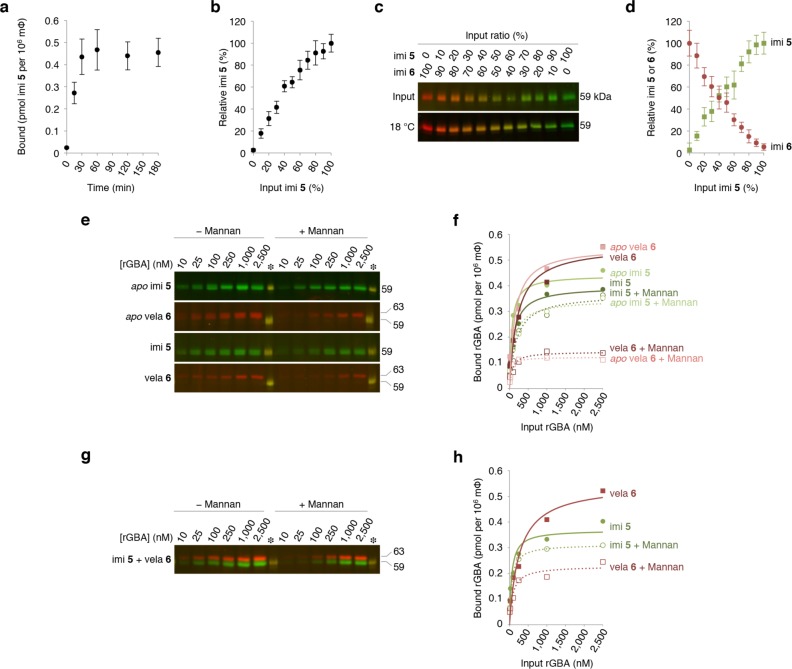
Binding of ABP-labeled imiglucerase to macrophages. (**a**) Time-dependent binding of green β-epoxide ABP **5**-labeled imiglucerase to macrophages at 18°C. (**b**) Dose dependence of binding of green β-epoxide ABP **5**-labeled imiglucerase to macrophages at 18°C. (**c**) Binding of mixtures of green ABP **5**− and red ABP **6**-labeled imiglucerase. (**d**) Quantification of **c**: cell-bound green ABP **5**-imiglucerase at 18°C related to input (*green closed square*); cell-bound red ABP **6**-imiglucerase at 18°C related to input (*red closed circle*). Data are average of duplicate experiments, ± SD. Effect of mannan on binding of ABP-labeled rGBAs. (**e**) Binding of 10−2,500 nM imiglucerase and velaglucerase at 18°C in the presence and absence of mannan (10 μg μL^−1^) shown for human monocyte-derived macrophages from one random donor (*Donor III*). From *top* to *bottom*: unlabeled (*apo*) imiglucerase post-labeled with green ABP **5**; *apo* velaglucerase post-labeled with red ABP **6**; pre-labeled green ABP **5** imiglucerase and pre-labeled red ABP **6**-velaglucerase. Fluorescence was calibrated with 50 fmol equimolar ABP **5**− and **6**-labeled imi present on each SDS-PAGE gel (*asterisk*). (**f**) Quantification of **e**; see S3 Fig for binding data of *n* = 12 donors. (**f**) Binding of equimolar 10−2,500 nM imiglucerase and velaglucerase at 18°C in the presence and absence of mannan (10 μg μL^−1^) shown for human monocyte-derived macrophages from one donor with green ABP **5**-labeled imiglucerase and red ABP **6**-labeled velaglucerase. Fluorescence was calibrated with 50 fmol equimolar ABP **5**− and **6**-labeled imi present on each SDS-PAGE gel (*asterisk*). (**h**) Quantification of **g**; see S4 Fig for binding data of *n* = 12 donors.

To determine binding, we firstly incubated human monocyte-derived macrophages with 100 nM green β-epoxide ABP **5**-labeled imiglucerase at 18°C. As shown in Fig 4A, binding was saturated at around 30 minutes. Enzyme binding was dose-dependent in the range 0–100 nM labeled rGBA (Fig 4B). Comparison of binding of green β-epoxide ABP **5**– and red ABP **6**-labeled imiglucerase concomitantly administrated (Fig 4C *and* 4D) indicated the binding affinity of the two ABP-labeled rGBAs is similar and thus binding is not influenced by the fluorophore incorporated into the ABP.

Uptake of ABP-labeled rGBA at 37°C was proportional to binding at 18°C (*see* S2 Fig). Incubation of the same cells with 100 nM green β-epoxide ABP **5**-labeled imiglucerase at 37°C showed that uptake was linear over time, up to 3 hours (S2 Fig). The same was observed for incubation of cells with mixtures of green ABP **5**− and red ABP **6**-labeled imiglucerase (*see* S2 Fig). Similar observations were made for binding and uptake by using unlabeled, *i*.*e*. *apo*, imiglucerase and ABP **5**-labeled imiglucerase (*Data not shown*).

Next, we analyzed the effect of mannan (10 μg μl^−1^) on binding and uptake of ABP-labeled rGBAs. Experiments were performed with macrophages differentiated monocytes isolated from blood samples of twelve healthy human donors. Binding of *apo* rGBAs was determined in the absence and presence of mannan. For this, cells were prior treated with excess CBE **3** for 2 hours to block the E340 nucleophiles of all endogenous GBA, next cells were washed and finally incubated at 18°C for 30 minutes with *apo* 10–2,500 nM imiglucerase or velaglucerase in the absence or presence of mannan (Fig 4E). Cell-bound rGBA was visualized by labeling with fluorescent ABPs: green β-epoxide ABP **5** in the case of imiglucerase and red β-epoxide ABP **6** in the case of velaglucerase. As can be seen in Fig 4E (*upper panels*), the presence of mannan hardly competed imiglucerase binding, but prominently inhibited surface binding of velaglucerase. The same observations were made with pre-labeled imiglucerase and velaglucerase (Fig 4E, *lower panels*). Binding of the former was hardly competed by mannan, but not that of pre-labeled velaglucerase (S3 Fig).

For fluorescence quantification and apparent K_D_ en B_MAX_ constants, see Fig 4F and Table 1, respectively. Similar trends were noted for cells of various donors. It can be seen in S7 Fig (*ratios of imiglucerase versus donors velaglucerase*) that inter-individual differences were noted in mannan-competed and non-competed binding of rGBAs. However, a larger mannan-competed binding of velaglucerase was always observed, as well as a larger mannan non-competed binding of imiglucerase. The finding that *apo* and pre-labeled rGBAs behaved entirely identical (*see* Fig 4E and 4F and Table 1) demonstrates that attachment of a β-epoxide ABP to the nucleophile E340 in the catalytic pocket does not influence recognition of enzyme by receptors involved in binding and uptake.

**Table 1:** Binding parameters.

	Mannan			
	Competed		Non-competed	
rGBA	Apparent K_D_	B_MAX_	Apparent K_D_	B_MAX_
**imiglucerase**	36.87	0.087	111.1	0.397
**velaglucerase**	359.9	0.434	215.9	0.556
**Ratio**	0.102	0.200	0.515	0.713

Apparent K_D_ and B_MAX_ of mannan competed− and non-competed binding. Effect of mannan on binding of equimolar ABP-labeled imiglucerase and velaglucerase.

The same experiments were performed while incubating cells with equimolar green β-epoxide ABP **5**-labeled imiglucerase and red ABP **6**-labeled velaglucerase (Fig 4G *and* 4H). Again, in this direct comparison of the two rGBAs, the mannan-competed binding was highest for velaglucerase (*see also* S7 Fig).

**Fig 5:**
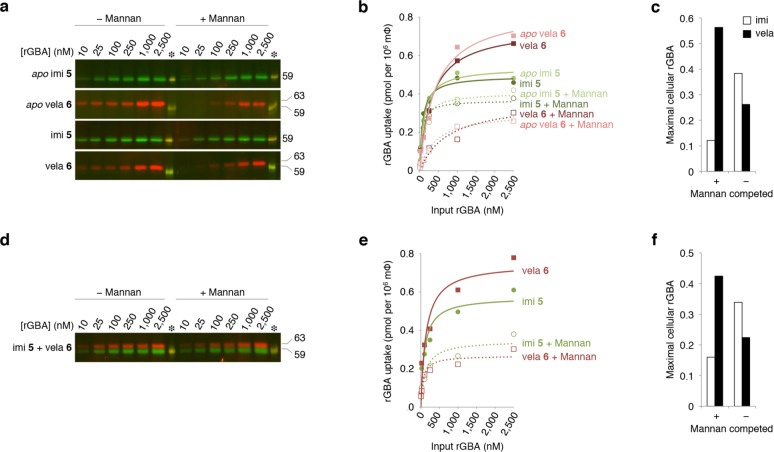
Effect of mannan on uptake of ABP-labeled rGBAs. (**a**) Binding and uptake of 10−2,500 nM imiglucerase and velaglucerase at 37°C in the presence and absence of mannan (10 μg μL^−1^) shown for human monocyte-derived macrophages from Donor III (*at random*, *see results* **Fig 4**). From *top* to *bottom*: unlabeled (*apo*) imiglucerase post-labeled with green ABP **5**; *apo* velaglucerase post-labeled with red ABP **6**; pre-labeled green ABP **5** imiglucerase and pre-labeled red ABP **6**-velaglucerase. Fluorescence was calibrated with 50 fmol equimolar ABP **5**− and **6**-labeled imi present on each SDS-PAGE gel (*asterisk*). (**b**) Quantification of **a**, subtracted was the binding observed at 18°C (*see* **Fig 4F**). See S5 Fig for uptake data of *n* = 12 donors. (**c**) Maximal cellular imiglucerase (*open column*) and velaglucerase (*closed column*) competed and non-competed by mannan. (**d**) Binding and uptake of equimolar 10−2,500 nM imiglucerase and velaglucerase at 37°C in the presence and absence of mannan (10 mg/mL) shown for human monocyte-derived macrophages from one donor with green ABP **5**-labeled imiglucerase and red ABP **6**-labeled velaglucerase. Fluorescence was calibrated with 50 fmol equimolar ABP **5**− and **6**-labeled imi present on each SDS-PAGE gel (*asterisk*). (**e**) Quantification of **d,** subtracted was the binding at 18°C (*see* **Fig 4I**). See S5 Fig for uptake data of *n* = 12 donors. (**f**) Maximal cellular imiglucerase (*open column*) and velaglucerase (*closed column*), competed and non-competed by mannan.

The effect of mannan on uptake of rGBAs by monocyte-derived macrophages was compared next. Hereto, human monocyte-derived macrophages were assayed with the exact same protocol (*vide supra*), except that incubation with the different enzyme mixtures occurred for 30 min at 37°C, allowing endocytosis to take place. As shown in Fig 5A and 5B, uptake of velaglucerase and imiglucerase, either *apo* or ABP-labeled, was different. Similar to the difference noted with binding, uptake competed by mannan was highest for velaglucerase, and uptake not competed by mannan was highest for imiglucerase. This is depicted also in Fig 5C for cells from identical donor as highlighted in Fig 4. Similar trends were seen for cells from other donors (*see* S8 Fig). Very similar findings were made when cells were exposed to mixtures of pre-labeled imiglucerase and velaglucerase (*see* Fig 5D–5F; *also* S9 Fig and S10 Fig).

### Bodily distribution of ABP-labeled imiglucerase and velaglucerase

**Fig 6:**
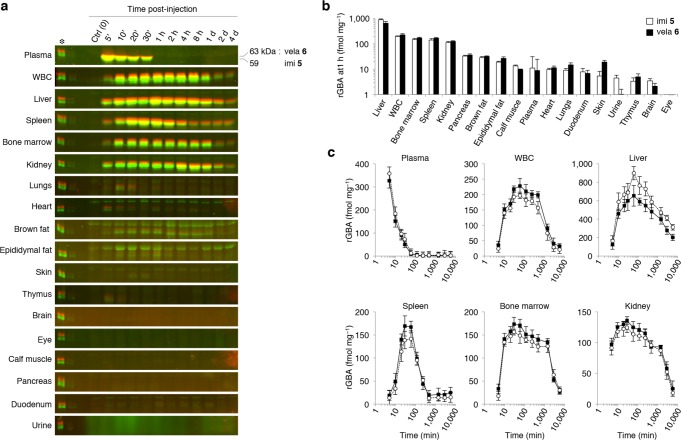
Bio-distribution of ABP-labeled rGBAs in living mice at different time-points. Wild-type male C57Bl/6J mice were injected with equimolar mixture of 1 nmol green β-epoxide ABP **5**-labeled imiglucerase (*imi*) and red β-epoxide ABP **6**-labeled velaglucerase (*vela*). The animals were sacrificed after 5, 10, 20, 30, 60, 120, 240, 480 minutes and 1, 2 and 4 days. (**a**) SDS-PAGE of tissues, with WBC as white-blood cells. Per tissue, equal amounts (10 μg) of protein were applied in every lane. Fluorescence was calibrated with 50 fmol equimolar green β-epoxide ABP **5**-labeled imiglucerase and red β-epoxide ABP **6**-labeled velaglucerase present on each SDS-PAGE gel (*asterisk*). (**b**) Tissue-level of ABP-labeled rGBA at one hour post-injection, ranked highest to lowest. ABP **5**-labeled imiglucerase (*open columns*) and ABP **6**-labeled velaglucerase (*closed columns*). (**c**) Quantification of ABP-fluorescence from **a**: ABP **5**-labeled imiglucerase (*open circles*) and ABP **6**-labeled velaglucerase (*closed squares*).

Finally, we studied the bio-distribution of imiglucerase and velaglucerase in living mice, by injecting twelve male, GBA wild-type (C56Bl6/J) mice intravenously in the tail vein with PBS vehicle or an equimolar mixture of 1 nanomole green β-epoxide ABP **5**-labeled imiglucerase and red β-epoxide ABP **6**-labeled velaglucerase. The animals were sacrificed after 5, 10, 20, 30, 60, 120, 240, 480 minutes and 1, 2 and 4 days post-injection with the ABP-labeled rGBA mixture. At each time-point, mice were anesthetized where after EDTA blood was taken *via* the tail vein, and urine and various tissues were collected following a perfusion *via* the heart with PBS. Tissue homogenates were prepared, equal amounts (10 μg total protein) were separated on SDS-PAGE gels in order to resolve the resident levels of fluorescent ABP-labeled imiglucerase and velaglucerase, and detected *via* fluorescence scanning of the slab-gel (Fig 6A).

The experiment revealed the half-life of both labeled rGBAs in plasma is approximately 10–15 minutes (Fig 6A, *top row*). Quantification of the ABP-emitted fluorescence, revealed the concentrations of both rGBAs were highest in the liver (*see* Fig 6B), followed by white-blood cells (WBC), bone marrow, spleen and kidney. With the mass of each isolated tissue, we determined most of the administered rGBA dose was recovered in the liver (88−93%), spleen (1.9−2.6%) and bone marrow (1.8−2.3%). In other organs, far less enzyme per amount of total protein was found, including the lungs. Barely detectable levels of enzyme were detected in brain and eye, which correlates with previous reports (*see* Fig 6B).

Importantly, no significant differences between imiglucerase and velaglucerase were observed, albeit marginally more imiglucerase localized in the liver than velaglucerase, while increased levels of the latter were identified in WBC, spleen and bone marrow.

Within approximately one hour post-injection, the amount of rGBAs per tissue reached its maximum. The apparent half-life of both labeled rGBAs was tissue-dependent (Fig 6C). A bi-phasic decay was noted for each investigated tissue, with generally a shorter half-life of about ~60−90 min and a much longer one (~12−18 hours, or more).

## Discussion

A rational concept for therapy of Gaucher disease is the chronic supplementation of lysosomes of storage macrophages with GBA.

To this end, the *N*-linked glycans of therapeutic GBAare modified to expose terminal mannose-moieties allowing uptake by mannose-receptor rich macrophages. Chronic intravenous administration of mannose-terminated GBA results in impressive clinical responses in type I GD patients. This has stimulatedcomparable enzyme therapy approaches for the treatment of other inherited lysosomal storage disorders such as Pompe disease [52], Fabry disease [53] and MPS disorders [54–56]. Enzyme therapy of type I GD presently employs recombinant glucocerebrosidases (rGBAs) produced in different platforms: imiglucerase, velaglucerase and taliglucerase. The various rGBAs all contain mannose-terminated *N*-linked glycans, but differences exist in the exact structures ranging from Man(3), Man(9) to plant-type high-mannose glycans. There is a present need for sensitive and comparative monitoring of the various therapeutic enzymes in pre-clinical and clinical studies. Particularly required is a method for non-invasive monitoring of tissue-distribution of rGBAs in individual type I GD patients. In connection to this, a new methodology for ultra-sensitive visualization of GBA has recently been developed. GBA can be subtly labeled through covalent attachment of fluorescent activity-based, cyclophellitol β-epoxide type, probes (ABPs) to its catalytic nucleophile E340. We have exploited such ABPs for comparative investigations of the two widely applied rGBAs in Europe: imiglucerase (Genzyme) and velaglucerase (Shire PLC).

Our investigation demonstrates that imiglucerase and velaglucerase can be conveniently labeled with fluorescent ABPs. ABP labeled rGBAs were found to behave similar to unlabeled enzyme in binding and uptake experiments with cultured denditric cells and macrophages. Of note, earlier non-specific labeling of rGBAs either by iodination or cross-linking fluorescent Oregon Green or Alexa Fluor was also reported not to affect binding and uptake [45, 57]. Since the enzymes can be selectively tagged with red− and green BODIPY fluorophores, mixtures of differently labeled rGBAs can be used for paired measurements and evaluation. When administered to cells expressing mannose-binding lectin(s), both rGBAs are avidly endocytosed, and this process is partly competed by mannan. Imiglucerase and velaglucerase concomitantly administered at equal dose to mice by intravenous infusion show a comparable tissue-distribution. The uptake of mannose-terminated rGBAs is thought to be mediated by the mannose-receptor [4, 16–18, 30, 32, 42–47, 57–64]. However, in recent years it has become apparent that multiple mannose-binding lectins exist in man. Besides the mannose receptor, also DC-SIGN, langerin, dectin 2 and others can potentially bind terminal mannose-containing structures [64–66]. The specificity of these various receptors for particular *N*-linked glycans is not well documented. Information is also limited regarding cell-type specific expression of the various receptors in man [67, 68]. Imiglucerase, velaglucerase and taliglucerase differ in *N*-linked glycan structures (*for an overview see* Fig 1A, Bruhmstein *et al*. [30] *and* Tekoah *et al*. [32]). It is therefore *a priori* unclear which mannose-binding lectins are responsible for binding and uptake of the various rGBAs in GD patients. Of interest, Gaucher cells in spleen of type I GD patients have been shown to express very little of the conventional mannose-receptor [69].

In a number of studies, binding and uptake of GBA by macrophages was investigated [28, 30, 32, 42, 43, 45, 46, 57–59, 61–63]. Unfortunately, different GBA preparations (alglucerase, imiglucerase, velaglucerase and taliglucerase) at different doses were incubated with different cells, *e*.*g*. differentiated cell-lines like U937, alveolar and peritoneal macrophages from mice and rats, as well as human monocyte-derived macrophages, hampering a direct comparison of reported data.

We comparatively studied binding and uptake of ABP-labeled imiglucerase and velaglucerase in cultured cells, including paired measurements. First we investigated the uptake of both rGBAs inmurine dendritic cells, showing comparable mannan-competitive uptake with continuous co-localization of both enzymes in intracellular structures. Next, we used human monocyte-derived macrophages as cell model system, allowing us to determine inter-individual differences. The initial rate of uptake of rGBAs by culture human monocyte-derived macrophages is impressive. When incubating monocyte-derived macrophages with 100 nM ABP-labeled imiglucerase or velaglucerase, cells endocytosed ~0.8 pmol GBA per million cells. For comparison, the amount of endogenous GBA in the same macrophages is an estimated ~0.25 pmol GBA per million cells. At a non-saturating concentration of 100 nM, small differences were noted between imiglucerase and velaglucerase: the binding and uptake of velaglucerase was slightly higher as observed with cells from twelve distinct healthy donors. Sato and Beutler also noted two distinct mannose-dependent uptake mechanisms for alglucerase in human monocyte-derived macrophages [57]. The K_D_ of the high-affinity mechanism was 120 nM [57]. Friedman *et al*. studying rat alveolar macrophages observed a K_D_ for mannan-competed binding of alglucerase of 13 nM [62]. In our cell model, the K_D_ of mannose-dependent binding was approximately ~88 nM for imiglucerase and ~162 nM for velaglucerase, probably reflecting a mixture of different mechanisms. Of further note, binding and uptake of rGBAs was only partly dependent on mannose-binding lectins. Particularly in the case of imiglucerase, significant binding and uptake still occurred in the presence of mannan. Recently the membrane protein LIMP-2 has been identified as a candidate receptor in glycan-independent binding and uptake of rGBA by blood cells [70]. LIMP-2 is already known to govern transport of newly formed GBA from the endoplasmic reticulum to the lysosome [71].

The relevance of the findings made with cultured macrophages for uptake of therapeutic enzyme in patients is still unclear. The bio-distribution of rGBAs has been studied in rodents. In rats, infusion of alglucerase was found to result in prominent delivery of enzyme to the liver [59, 60]. In this tissue, most of the enzyme was recovered in sinusoidal endothelial cells and in Kupffer cells. Hepatic sinusoidal endothelial cells are known to express the mannose-receptor [59, 72–74], explaining their prominent uptake of mannose-terminated GBA. Uptake of mannose-terminated GBA by hepatic sinusoidal endothelial cells was further confirmed in later studies [46, 58, 62]. The half-life of alglucerase in plasma of rats was found to be remarkably short, around 2–5 minutes [59], mimicking the values of 3–12 min observed in type I GD patients (Aerts *et al*., *unpublished observations*). Similar findings have been made with mice [43]. A large portion of infused alglucerase was recovered in the liver (60%) and spleen (20%) [43]. Maximal amounts were noted about 20 minutes post-infusion. More recently, Shaaltiel and co-workers [32] reported on the comparison between imiglucerase, velaglucerase and taliglucerase each administered to mice at a dose of 90 IU kg^–1^. Similar findings were made regarding bio-distribution, which was identical for all three rGBAs. van Patten *et al*. [45] produced in various expression systems rGBAs with different mannose-type *N*-linked glycans ranging from Man(2) to Man(9). No difference was observed among the enzymes regarding affinity to the mannose-receptor, macrophage uptake and intracellular half-life. In a Gaucher mouse model (D409V/null), neither striking differences were seen for clearance or tissue-distribution of the various rGBAs [45]. Our present study with ABP-labeled imiglucerase and velaglucerase recapitulates the earlier findings in many aspects. We administered to mice 1 nanomol (3 IU) of ABP-labeled imiglucerase and velaglucerase each (*i*.*e*. 100 IU kg^–1^), mimicking enzyme doses administered to human Gaucher patients (30–60 IU kg^–1^). Both administered enzymes showed a short plasma half-life (10–15 min). Liver (88−93%), spleen (1.9−2.6%) and bone marrow (1.8−2.3%) contained most of the infused enzyme, both with imiglucerase and velaglucerase. The amount of both rGBAs was maximal in most tissues at 60 minutes post-infusion.

The animal studies rendered some insight in stability of infused enzyme. Murray and co-workers [60], and independently Xu and colleagues [43], reported a bi-phasic decay of alglucerase with a short half-life of 45 min and a longer one of 12 hours. Shaaltiel and co-workers [32], studying intravenous infusions of imiglucerase, velaglucerase and taliglucerase in mice, observed for all three rGBAs a rapid decay of tissue enzyme activity, being around 50% at 60 min post-infusion as compared to the maximal level at 20 minutes post-infusion [32]. Of interest, the half-life for endogenous GBA in cultured fibroblasts is known to be significantly longer, being around two days. It is unclear whether the rapid decrease of rGBA in tissues noted in various studies is due to regular proteolytic breakdown in lysosomes. Alternatively, the administered exogenous enzyme could be handled differently from normal endogenous GBA that reaches lysosomes bound to LIMP-2. It cannot be excluded that exogenous GBA is partly released from some cell types by endosomal or lysosomal exocytosis, or is subjected to specific degradation. A similar conclusion regarding a distinct fate of endogenous GBA and exogenous GBA was earlier drawn by Murray and co-workers [60]. Piepenhagen *et al*. [46] non-specifically labeled alglucerase and imiglucerase with Oregon Green 488 and Alexa Fluor 546 carboxylates using succinimidyl-esters [46]. Prominent delivery of tagged GBAs to Kupffer cells and sinusoidal endothelial cells in the livers of infused mice was observed. In our investigation we employed GBAs specifically labeled in their catalytic pocket. There is strong evidence that the lysosomal stability of GBA is markedly improved by such ABP labeling (Kallemeijn et al., submitted for publication). A similar phenomenon is observed with ABP labeling of the β-glucosidase EGCase II [75]. Infused ABP-labeled rGBAs in all tissues of mice showed a bi-phasic decay with a very long half-life (>12 hours), possibly partly due to ABP-mediated stabilization, and a much shorter one of ~60−90 min.

Information on the tissue-distribution and half-life of rGBA following infusion of GD patients is at present almost completely indirect. Clinical responses have been used to speculate on delivery of rGBA to particular tissues. Reductions in liver and spleen volumes are thought to reflect local supplementation with rGBA. Beneficial responses in hematological parameters and skeletal disease are believed to point to targeting of enzyme to the bone marrow. The lack of response in neurological manifestations to enzyme therapy is ascribed to inability of the rGBAs to reach neuronal cells. Only in a single type I GD patient, the tissue-distribution of ^123^I-modified placental GBA (alglucerase) was visualized. Delivery of enzyme to liver, spleen and bone marrow was demonstrated. Autopsy materials of two type II GD patients, treated with alglucerase 48 hours prior to death, have been examined suggesting delivery of enzyme to liver and spleen, but not to lung and brain [76]. Comparable data for the widely applied rGBAs are completely lacking.

Our investigation supports the notion that it is feasible to monitor in Gaucher patients the tissue-targeting of infused rGBAs pre-labeled with a fluorophore-containing ABP. We are presently designing ABPs bearing fluorophores suitable for whole-body *in vivo* detection of labeled enzyme *via* infrared imaging. Alternatively, other tags linked to the ABP may be considered to permit non-invasive whole body monitoring such as positron-emission tomography (PET) tags [77] or single photon emission computed tomography (SPECT) probes [78, 79]. Dual isotope SPECT methods (*for example combining* ^177^Lu *and* ^111^In*-labels*) would allow even simultaneous *in vivo* determination of the bio-distribution of two different recombinant enzymes [79]. With such methods in place it should become possible to study in more detail the targeting of the present rGBAs with distinct mannose-terminated *N*-linked glycans.

## Conclusion

Fluorescent activity-based probes can be used to label different rGBAs in a defined manner, allowing the simultaneous monitoring of binding of various rGBA types onto the cellular surface of macrophages and uptake therein, even tissue-distribution in laboratory animals (Fig 6). We noted slight differences between the rGBA preparations of imiglucerase and velaglucerase, in terms of their binding and uptake within monocyte-derived macrophages, along with their in vivo biodistribution, however direct paired microscopic analyses did not reveal differences in their cellular localization. The technological approach could be further developed into a procedure for non-invasive monitoring of whole-body distribution of rGBAs using trace amounts of labeled enzyme with a tailor-made tag for detection.

## Supporting information

S1 Fig *In vitro* characterization of rGBAs. (DOCX)

S2 Fig Dose-dependence and proportional binding and uptake of ABP-labeled imiglucerase. (DOCX)

S3 Fig Binding of ABP-labeled rGBAs to macrophages. (DOCX)

S4 Fig Binding of equimolar ABP-labeled rGBAs to macrophages. (DOCX)

S5 Fig Uptake of ABP-labeled rGBAs to macrophages. (DOCX)

S6 Fig Uptake of equimolar ABP-labeled rGBAs to macrophages. (DOCX)

S7 Fig Mannan-competed and non-competed binding of imiglucerase and velaglucerase after separate incubation of macrophages with enzyme. (DOCX)

S8 Fig Mannan-competed and non-competed binding to cultured macrophages after incubation with equimolar mixture of imiglucerase and velaglucerase. (DOCX)

S9 Fig Mannan-competed and non-competed cellular imiglucerase and velaglucerase after separate incubation of macrophages with each enzyme. (DOCX)

S10 Fig Mannan-competed and non-competed cellular rGBA after incubation of macrophages with equimolar mixture of imiglucerase and velaglucerase. (DOCX)

## Abbreviations

ABP: activity-based probe
AMP-DNM: *N*-(5-adamantane-1-*yl*-methoxy)pentyl)-deoxynojirimycin
CBE: conduritol beta-epoxide
ERT: enzyme replacement therapy
(r)GBA: (recombinant) glucocerebrosidase
GD: Gaucher disease
imi: imiglucerase
vela: velaglucerase
