# Supplementary material for: Investigations on therapeutic glucocerebrosidases through paired detection with fluorescent activity-based probes
Source: PLoS One. 2017 Feb 16;12(2):e0170268. doi: 10.1371/journal.pone.0170268 (PMC5313132; doi:10.1371/journal.pone.0170268)
Supplement: S1 Fig — (DOCX) [file pone.0170268.s001.docx]

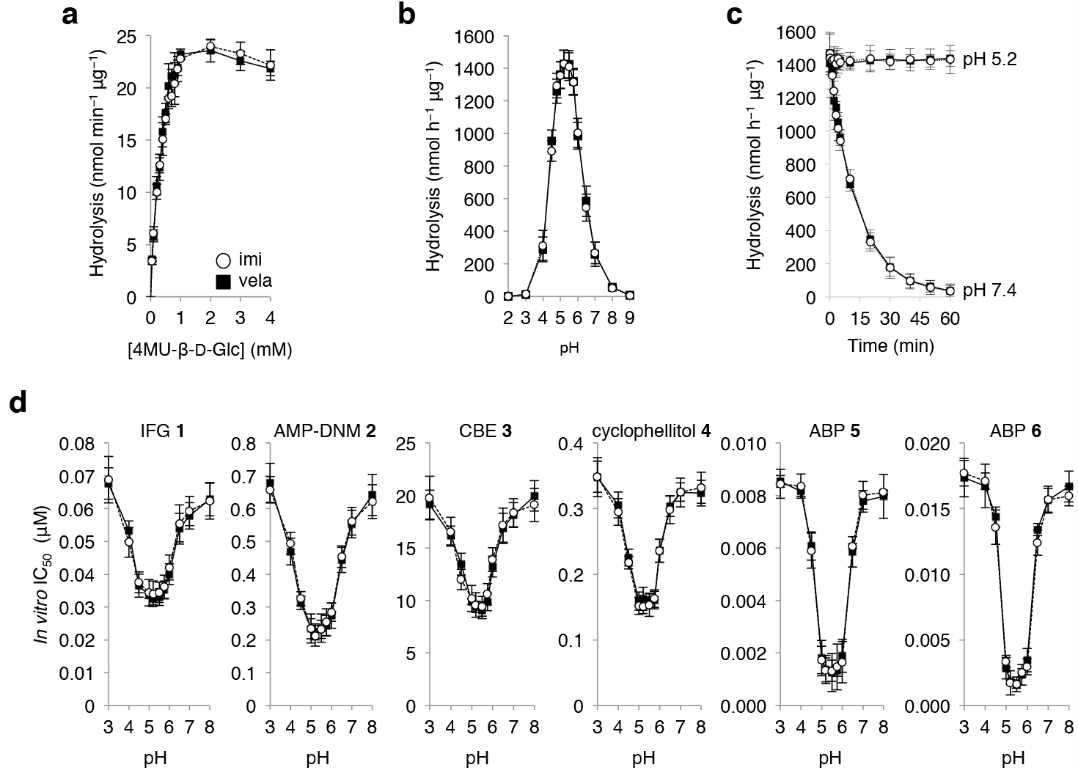


**S1 Figure 1 | *In vitro* characterization of rGBAs. (a**) Hydrolysis of artificial 4MU-β-d*-*Glc at pH 5.2 as described in **Experimental Section** by imiglucerase (*open circles*) and velaglucerase (*closed squares*). Data are average of duplicate experiments, ± SD. (**b**) Effect of pH on 4MU-β-d*-*Glc hydrolysis. Data are average of duplicate experiments, ± SD. (**c**) Half-life of active enzyme at pH 7.4 and pH 5.2. Data are average of duplicate experiments, ± SD. (**d**) Influence of pH on the inhibitory potential (*half-maximal inhibitory concentration*; IC_50_) of IFG **1**, AMP-DNM **2**, CBE **3**, cyclophellitol **4**, ABP **5** and **6**. Data are average of triplicates, ± SD.
