# Supplementary material for: Investigations on therapeutic glucocerebrosidases through paired detection with fluorescent activity-based probes
Source: PLoS One. 2017 Feb 16;12(2):e0170268. doi: 10.1371/journal.pone.0170268 (PMC5313132; doi:10.1371/journal.pone.0170268)
Supplement: S2 Fig — (DOCX) [file pone.0170268.s002.docx]

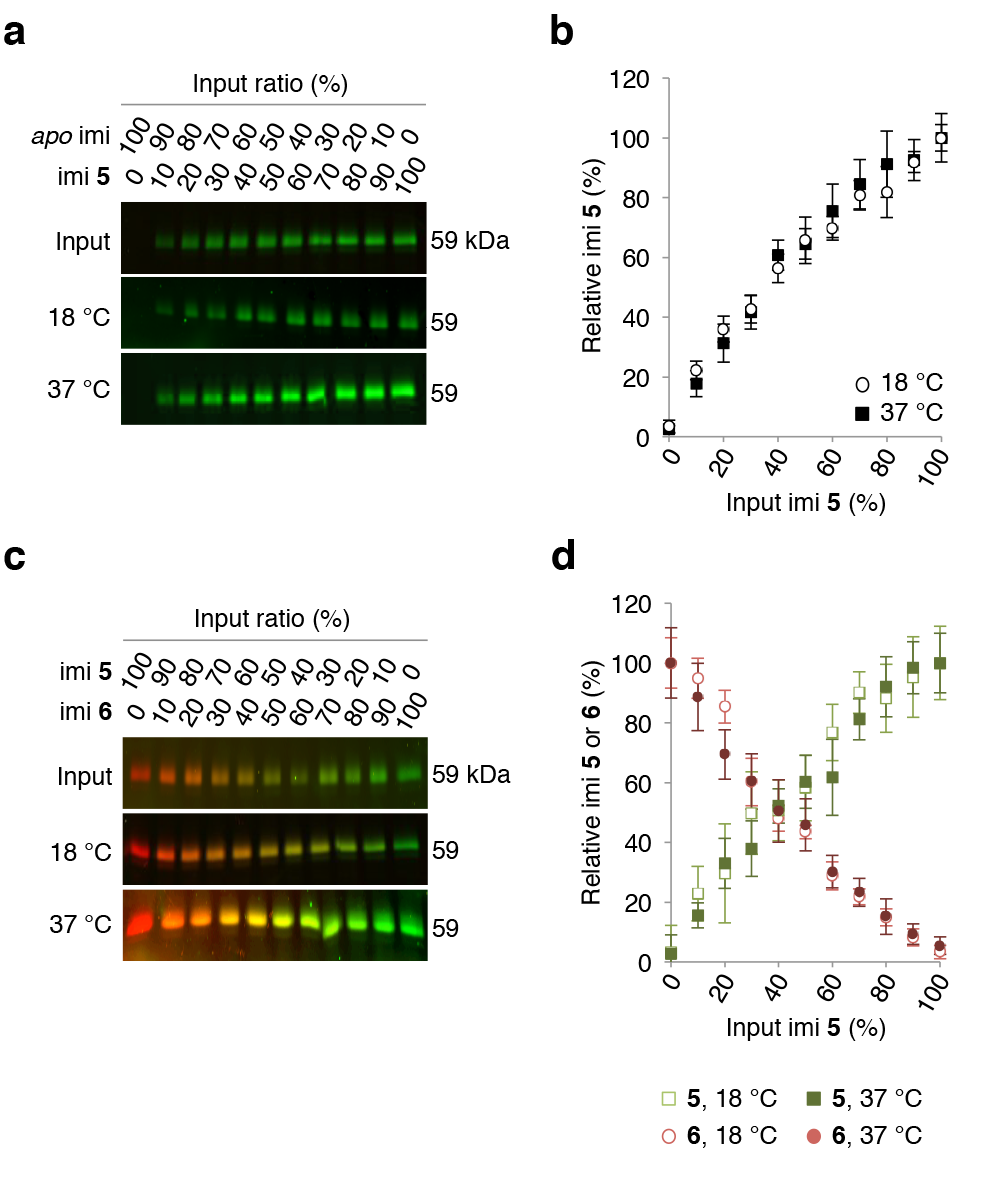


**S1 Figure 2 | Dose-dependence and proportional binding and uptake of ABP-labeled imiglucerase.** (**a**) Human monocyte-derived macrophages were incubated with increasing ABP **5**-labeled imi (*top*) at 18 °C (*binding*) or 37 °C (*binding* + *uptake*). (**b**) Quantification of **a**: cell-bound imiglucerase at 18 °C related to input (*open circle*) and cell-bound imiglucerase at 37 °C related to input (*closed square*). Data are average of duplicate experiments, ± SD. (**c**) Similar to **a**: except exposure of cells to mixture of green ABP **5**− and red ABP **6**-labeled imiglucerase. (**d**) Idem **b** for mixture of green ABP **5**− and red ABP **6**-labeled imiglucerase: cell-bound green ABP **5**-imiglucerase at 18 °C related to input (*green closed square*); cell-bound red ABP **6**-imiglucerase at 18 °C related to input (*red closed circle*); cell-bound green ABP **5**-imiglucerase at 37 °C related to input (*green* *open square*); cell-bound red ABP **6**-imiglucerase at 37 °C related to input (*red* *open circle*). Data are average of duplicate experiments, ± SD.
