# Supplementary material for: Investigations on therapeutic glucocerebrosidases through paired detection with fluorescent activity-based probes
Source: PLoS One. 2017 Feb 16;12(2):e0170268. doi: 10.1371/journal.pone.0170268 (PMC5313132; doi:10.1371/journal.pone.0170268)
Supplement: S6 Fig — (DOCX) [file pone.0170268.s006.docx]

**
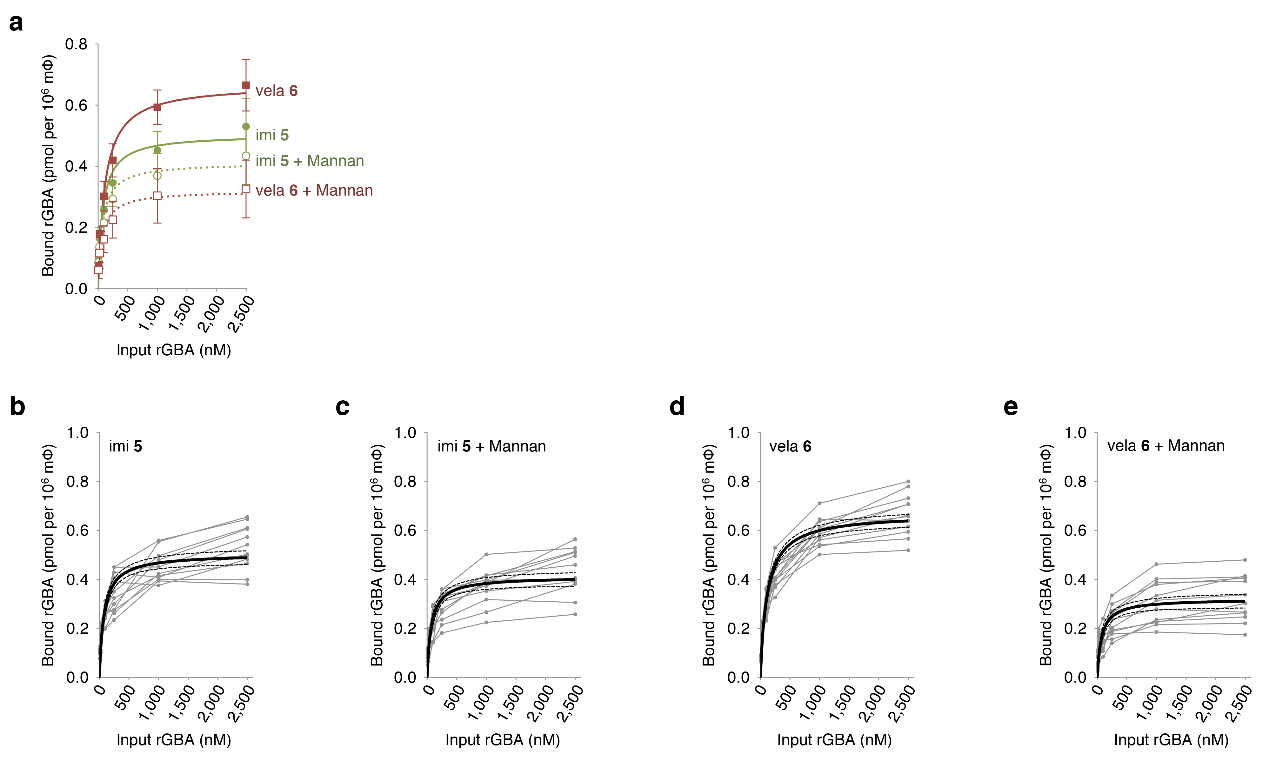
**

**S1 Figure 6 | Uptake of equimolar ABP-labeled rGBAs to macrophages.** (**a**) Uptake of equimolar 10−2,500 nM imiglucerase and velaglucerase at 18 °C in the presence and absence of mannan (10 μg μL^−1^) for human monocyte-derived macrophages. Data are average of *n* = 12, ± SD. Individual experiments are depicted in **b**-e, with the solid line representing the average and dotted line the 95% confidence interval.
