# Supplementary material for: Investigations on therapeutic glucocerebrosidases through paired detection with fluorescent activity-based probes
Source: PLoS One. 2017 Feb 16;12(2):e0170268. doi: 10.1371/journal.pone.0170268 (PMC5313132; doi:10.1371/journal.pone.0170268)
Supplement: S7 Fig — (DOCX) [file pone.0170268.s007.docx]

**
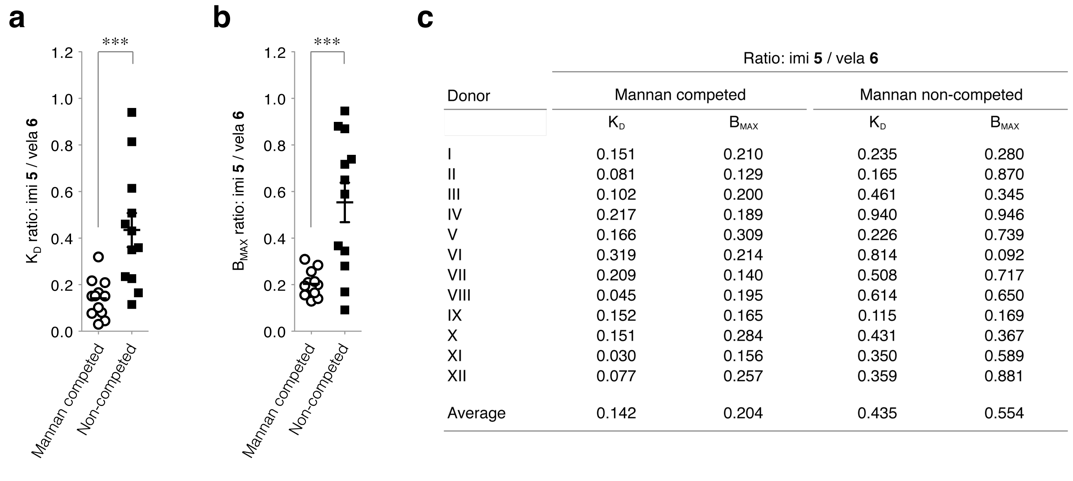
**

**S1 Figure 7 | Mannan-competed and non-competed binding of imiglucerase and velaglucerase after separate incubation of macrophages with enzyme.** (**a**) K_D_ observed with cells from twelve healthy donors. Expressed as ratio of imiglucerase/velaglucerase. Experiment performed in the presence and absence of mannan, for twelve healthy donors. (**b**) B_MAX_, *vide supra*. (**c**) K_D_ and B_MAX_ observed for cells from each separate healthy donor. All data are average of duplicate analyses per donor, with Student *t*-test significance *p* < 0.001***.
