# Supplementary material for: Investigations on therapeutic glucocerebrosidases through paired detection with fluorescent activity-based probes
Source: PLoS One. 2017 Feb 16;12(2):e0170268. doi: 10.1371/journal.pone.0170268 (PMC5313132; doi:10.1371/journal.pone.0170268)
Supplement: S9 Fig — (DOCX) [file pone.0170268.s009.docx]

**
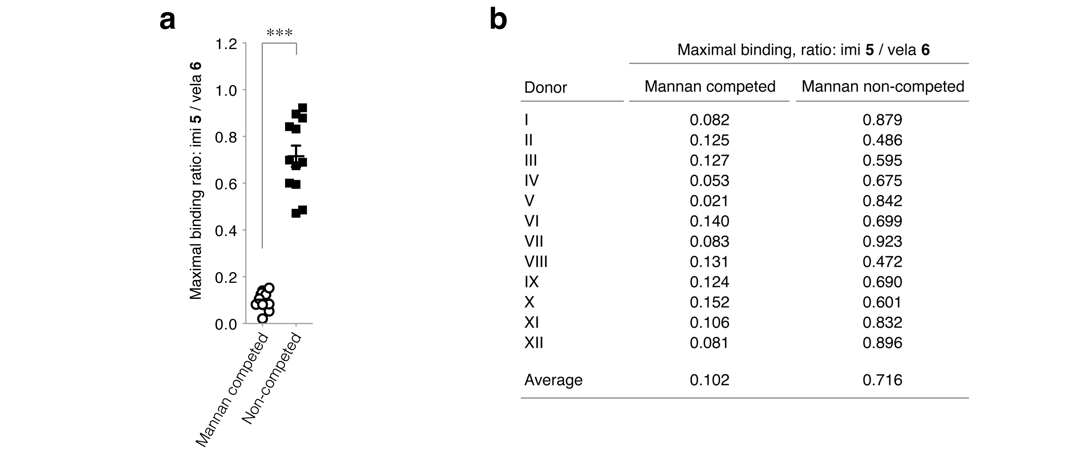
**

**S1 Figure 9 | Mannan-competed and non-competed cellular imiglucerase and velaglucerase after separate incubation of macrophages with each enzyme.** (**a**) Maximal cellular rGBA in cultured macrophages obtained from twelve healthy donors. Expressed as ratio of imiglucerase/velaglucerase. Experiments performed in absence and presence of mannan. (**b**) Maximal cellular rGBA observed with cells from each separate healthy donor. All data are average of duplicate analyses per donor, with Student t-test significance *p* < 0.001***.
