# Supplementary material for: Investigations on therapeutic glucocerebrosidases through paired detection with fluorescent activity-based probes
Source: PLoS One. 2017 Feb 16;12(2):e0170268. doi: 10.1371/journal.pone.0170268 (PMC5313132; doi:10.1371/journal.pone.0170268)
Supplement: S10 Fig — (DOCX) [file pone.0170268.s010.docx]

**
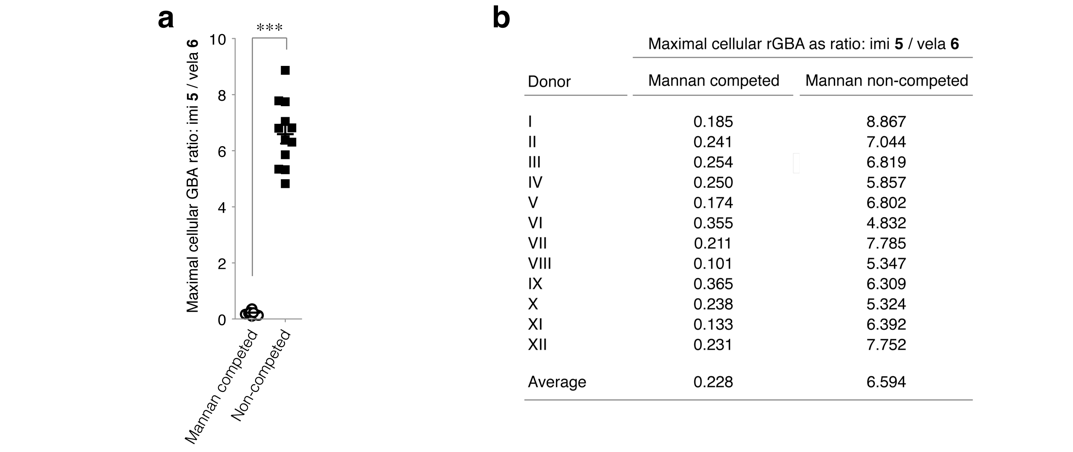
**

**S1 Figure 10 | Mannan-competed and non-competed cellular rGBA after incubation of macrophages with equimolar mixture of imiglucerase and velaglucerase.** (**a**) Maximal cellular rGBA in cultured macrophages obtained from twelve healthy donors. Expressed as ratio of imiglucerase/velaglucerase. Experiments performed in absence and presence of mannan.(**b**) Maximal cellular rGBA observed with cells from each separate healthy donor. All data are average of duplicate analyses per donor, with Student t-test significance *p* < 0.001***.
